# Prognostic factors in patients admitted to an urban teaching hospital with COVID-19 infection

**DOI:** 10.1186/s12967-020-02524-4

**Published:** 2020-09-15

**Authors:** Donogh Maguire, Marylynne Woods, Conor Richards, Ross Dolan, Jesse Wilson Veitch, Wei M. J. Sim, Olivia E. H. Kemmett, David C. Milton, Sophie L. W. Randall, Ly D. Bui, Nicola Goldmann, Allan Cameron, Barry Laird, Dinesh Talwar, Ian Godber, Alan Davidson, Donald C. McMillan

**Affiliations:** 1 Emergency Medicine Department, Glasgow Royal Infirmary, 84 Castle Street, Glasgow, G4 0SF UK; 2 School of Medicine Veterinary and Life Sciences, Wolfson Medical School Building, University of Glasgow, University Avenue, Glasgow, G12 8QQ UK; 3 Academic Unit of Surgery, School of Medicine, University of Glasgow, New Lister Building, Royal Infirmary, Glasgow, G31 2ER UK; 4 Department of Acute Medicine, Glasgow Royal Infirmary, Glasgow, G4 0SF UK; 5 Institute of Genetics and Molecular Medicine, University of Edinburgh, Edinburgh, EH4 2XU UK; 6 St Columba’s Hospice, 15 Boswall Rd, Edinburgh, EH5 3RW UK; 7 The Scottish Trace Element and Micronutrient Reference Laboratory, Department of Biochemistry, Royal Infirmary, Glasgow, G31 2ER UK; 8 Department of Clinical Biochemistry, Queen Elizabeth University Hospital, Govan, Glasgow, G51 4TF UK

**Keywords:** COVID-19, Systemic inflammatory response (SIRS), C-reactive protein (CRP), Albumin, Peri-operative glasgow prognostic score (poGPS), Neutrophil lymphocyte ratio (NLR), 30-day mortality, Host inflammatory response

## Abstract

**Background:**

Severe COVID-19 infection results in a systemic inflammatory response (SIRS). This SIRS response shares similarities to the changes observed during the peri-operative period that are recognised to be associated with the development of multiple organ failure.

**Methods:**

Electronic patient records for patients who were admitted to an urban teaching hospital during the initial 7-week period of the COVID-19 pandemic in Glasgow, U.K. (17th March 2020—1st May 2020) were examined for routine clinical, laboratory and clinical outcome data. Age, sex, BMI and documented evidence of COVID-19 infection at time of discharge or death certification were considered minimal criteria for inclusion.

**Results:**

Of the 224 patients who fulfilled the criteria for inclusion, 52 (23%) had died at 30-days following admission. COVID-19 related respiratory failure (75%) and multiorgan failure (12%) were the commonest causes of death recorded. Age ≥ 70 years (p < 0.001), past medical history of cognitive impairment (p ≤ 0.001), previous delirium (p < 0.001), clinical frailty score > 3 (p < 0.001), hypertension (p < 0.05), heart failure (p < 0.01), national early warning score (NEWS) > 4 (p < 0.01), positive CXR (p < 0.01), and subsequent positive COVID-19 swab (p ≤ 0.001) were associated with 30-day mortality. CRP > 80 mg/L (p < 0.05), albumin < 35 g/L (p < 0.05), peri-operative Glasgow Prognostic Score (poGPS) (p < 0.05), lymphocytes < 1.5 10^9^/l (p < 0.05), neutrophil lymphocyte ratio (p ≤ 0.001), haematocrit (< 0.40 L/L (male)/ < 0.37 L/L (female)) (p ≤ 0.01), urea > 7.5 mmol/L (p < 0.001), creatinine > 130 mmol/L (p < 0.05) and elevated urea: albumin ratio (< 0.001) were also associated with 30-day mortality.

On multivariate analysis, age ≥ 70 years (O.R. 3.9, 95% C.I. 1.4–8.2, p < 0.001), past medical history of heart failure (O.R. 3.3, 95% C.I. 1.2–19.3, p < 0.05), NEWS > 4 (O.R. 2.4, 95% C.I. 1.1–4.4, p < 0.05), positive initial CXR (O.R. 0.4, 95% C.I. 0.2–0.9, p < 0.05) and poGPS (O.R. 2.3, 95% C.I. 1.1–4.4, p < 0.05) remained independently associated with 30-day mortality.

Among those patients who tested PCR COVID-19 positive (n = 122), age ≥ 70 years (O.R. 4.7, 95% C.I. 2.0—11.3, p < 0.001), past medical history of heart failure (O.R. 4.4, 95% C.I. 1.2–20.5, p < 0.05) and poGPS (O.R. 2.4, 95% C.I. 1.1–5.1, p < 0.05) remained independently associated with 30-days mortality.

**Conclusion:**

Age ≥ 70 years and severe systemic inflammation as measured by the peri-operative Glasgow Prognostic Score are independently associated with 30-day mortality among patients admitted to hospital with COVID-19 infection.

## Background

As of 27th May 2020, approximately 5.7 million people worldwide are known to have been infected with COVID-19 coronavirus and more than 350,000 have died [1]. The severity of this viral disease for an individual is associated with a widespread perturbation of immune, physiological and metabolic parameters [2, 3]. These whole body changes could be considered characteristic of a systemic inflammatory response to tissue injury and it has been long recognised that a large and ongoing systemic inflammatory response is associated with the development of multiple organ failure and infective disease [4, 5].

One of the cardinal signs of severe COVID-19 infection is a marked systemic inflammatory response [2]. This response bears striking similarity to the systemic inflammatory response experienced by patients undergoing major elective surgical resections for cancer [6, 7]. Indeed, the systemic inflammatory response and the associated metabolic stress has been most well characterised in major elective surgery, where the relationship between the magnitude of the post-operative systemic inflammatory response and the development of post-operative complications is now well recognised, as is the effect of patient comorbidity on this relationship [8, 9]. Such work has informed therapeutic manoeuvres including minimally invasive surgery, pre-operative optimisation (e.g. anaesthesia, nutrition and steroids) and enhanced recovery protocols.

The aim of the present study was to examine whether routinely collected clinicopathological characteristics of patients with COVID-19 on admission were informative on the immune and metabolic stress experienced by patients with COVID-19 and whether such characteristics were informative on subsequent outcome.

## Patients and methods

Electronic patient records for patients who attended the Emergency Department (ED) and Acute Assessment Unit (AAU) at Glasgow Royal Infirmary (GRI), Glasgow, U.K., during the initial 7-week period of the COVID-19 pandemic in Glasgow city (17th March 2020–1st May 2020) were examined for routine clinical, laboratory and clinical outcome data. GRI is a university teaching hospital that serves an urban population with a high burden of socio-economic deprivation and offers the full spectrum of adult acute receiving specialties to patients over 16 years old. In line with NHS policy, this study was approved by the NHS Greater Glasgow and Clyde Caldicott guardian. The study protocol (GN20AE307) was approved by the North West England—Preston research ethics committee (20/NW/0336) and registered with clinicaltrials.gov (NCT04484545).

Patients displaying clinical signs or reporting symptoms consistent with possible COVID-19 infection (as defined by Health Protection Scotland) [10] at the time of presentation to ED and AAU were assessed for inclusion in the study. Patients who were reported by a board certified radiologist to have radiological changes characteristic of COVID-19 infection reported on chest X-ray (CXR) or CT thorax, were assessed for inclusion in the study. Patients who were admitted with other conditions and tested polymerase chain reaction (PCR) positive following admission were also included in the analysis. SARS-CoV-2 PCR testing was performed on all patients included in the sample, however only 122 of the 264 patients who satisfied HPS criteria for clinical diagnosis of SARS-CoV-2 and were admitted to hospital, subsequently had the diagnosis confirmed with positive SARS-CoV-2 PCR test. Age, sex, BMI and documented (clinical, radiological or PCR) evidence of COVID-19 infection at time of discharge or death certification were considered minimal criteria for inclusion.

As per routine clinical practice in the Emergency Department and Acute Assessment Area at GRI, patients were scored on the National Early Warning Score (NEWS) at presentation to triage. NEWS is a validated score of severity of physiological derangement that allocates a score (0–3) to six clinical parameters (pulse rate, blood pressure, respiratory rate, oxygen saturations, requirement for supplemental oxygen and level of responsiveness (alert (A), responding to verbal (V), painful (P) stimuli and unresponsive (U) (AVPU scale)) [11]. NEWS determines the triage category and level of immediate treatment that is required at the time of presentation, and the interval to re-administering the NEWS scoring tool according to the score achieved (i.e. the severity of physiological derangement). NEWS > 4 and > 7 are considered to indicate moderately severe and severe physiological derangement respectively.

Age was grouped as less than 40 years, 40–49 years, 50–59 years, 60–69 years, 70–79 years and 80 years and older. Age categories were further simplified to < / ≥ 70 years (see Tables 3, 4, 5). Social deprivation was defined by the Scottish Indices of Multiple Deprivation 2019 based on individual home postcode. Ethnicity was classified as White, Mixed, Asian, Black, or other ethnic group. Frailty was assessed using the Clinical Frailty Scale (CFS) [12, 13].

Admission serum C-reactive protein (CRP), albumin and differential blood cell counts were categorised using local reference intervals. Neutrophil/lymphocyte ratio (NLR) and the peri-operative Glasgow Prognostic Score (poGPS) were calculated as outlined in Tables 1, 2 [6, 14, 15]. The neutrophil lymphocyte ratio (NLR) is a validated prognostic scoring system that has been used in a variety of clinical settings. It utilises two components of the differential white cell count that are routinely measured in patients admitted to the general hospital setting. However, studies utilising the NLR in sepsis and peri-operative prognostic scores have used a variety of thresholds, making inter-study extrapolation of results difficult. For this study, thresholds of NLR ≤ 3, > 3—< 5 and ≥ 5 have been chosen, indicating mild, moderate and severe systemic inflammatory response respectively [16].

**Table 1:** Calculation of the Neutrophil Lymphocyte Ratio (NLR)

Neutrophil Lymphocyte Ratio (NLR):	Ratio	SIRS severity
Neutrophil count: lymphocyte count	< 3	Mild
Neutrophil count: lymphocyte count	3–5	Moderate
Neutrophil count: lymphocyte count	> 5	Severe

**Table 2:** Peri-operative Glasgow Prognostic Score (poGPS)

peri-operative Glasgow Prognostic Score (poGPS)	Score	SIRS severity
C-reactive protein ≤ 150 mg/l and Albumin ≥ 25 g/l	0	Mild
C-reactive protein > 150 mg/l and Albumin ≥ 25 g/l	1	Moderate
C-reactive protein ≤ 150 mg/l and Albumin < 25 g/l	1	Moderate
C-reactive protein > 150 mg/l and Albumin < 25 g/l	2	Severe

### Statistical analysis

Autobiographical data, clinicopathological data and haematological/biochemical results were presented as categorical variables. Categorical variables were analysed using χ^2^ test for linear-by-linear association, or χ^2^ test for 2-by-2 tables.

Associations between autobiographical data, clinicopathological characteristics, haematological/biochemical results and survival were analysed using univariate and a multivariate backward conditional approach. A *p* < 0.05 was applied to inclusion at each step in the multivariate analysis.

A convenience sampling strategy was adopted based on the patients admitted during the study period; therefore a formal sample size calculation was not performed. Missing data were excluded from analysis on a variable-by-variable basis. Two-tailed *p* values < 0.05 were considered statistically significant. Statistical analysis was performed using SPSS software version 25.0. (SPSS Inc., Chicago, IL, USA).

## Results

Of the 359 patients who attended Glasgow Royal Infirmary and satisfied HPS criteria for categorising as a possible COVID -19 related presentation, 241 patients fulfilled the criteria for inclusion with age, sex, BMI and documented evidence of COVID-19 infection at discharge or death certification. Seventeen patients were re-admitted and these were excluded from the analysis at second admission leaving 224 patients to be included in the analysis. The clinicopathological characteristics at presentation are shown in Table 3. The majority of patients were ≥ 70 years old (88%), male (55%), were not obese (57%) and were socioeconomically deprived (SE groups 1 and 2, 64%). The majority of patients were living independently in their own home (85%) and of white Scottish (93%) ethnicity. The majority of patients did not have comorbid disease including hypertension (60%), heart failure (90%) or type 2 diabetes (77%) and were not frail (54%). The median BMI was 29·0 kg/m^2^, with 33% of individuals having a BMI of less than 26 kg/m^2^, and 25% exceeding a BMI of 35.0 kg/m^2^.

**Table 3:** Univariate analysis of clinicopathological characteristics of patients admitted with symptoms of COVID-19 (n = 224)

	Alive (n = 172)	Dead (n = 52)	p-value
Age (< / ≥ 70 years)	124/48	19/33	< 0.001
Sex (male/female)	91/81	33/19	0.181
BMI (< 20; ≥ 20–29; ≥ 30 kg/m^2^)	13/85/74	4/25/23	0.867
SIMD (1 (most)–6 (least) deprived)	81/31/17/23/1/19	24/4/7/11/0/6	0.685
Ethnicity (1–5)	159/0/7/2/3	50/0/0/0/2	0.774
Living circumstances (0–3)	151/11/7/3	39/2/8/3	0.008
Past Medical History			
Hypertension (y/n)	63/109	28/24	0.027
Heart failure (y/n)	12/160	11/41	0.003
T1DM (y/n)	2/170	0/52	0.436
T2DM (y/n)	37/135	15/37	0.273
Chronic renal failure (y/n)	18/154	10/42	0.095
Cognitive impairment (y/n)	16/156	14/38	0.001
Previous delirium (y/n)	6/166	10/40	< 0.001
Frailty score (≤ / > 3)	107/65	14/38	< 0.001
COPD (y/n)	29/143	12/40	0.311
Smoker (never/ex/active)	82/70/20	24/27/1	0.428
Alcohol excess (y/n)	22/150	9/43	0.410
Liver disease (y/n)	15/157	4/48	0.816
Hep C (never/previous/active)	167/2/2	51/1/0	0.650
Active cancer (y/n)	6/166	3/49	0.464
Asthma (y/n)	42/130	4/48	0.009
Surgery < 1 yr (y/n)	17/154	4/44	0.277
Cancer resection (y/n)	1/171	0/52	0.582
Diagnostic criteria			
PCR positive/Clinical Dx/Radiological Dx	74/7/91	37/1/14	0.001
PCR negative/indeterminate/positive	43/42/83	4/9/39	0.001
CXR negative/positive	63/107	30/21	0.006
Physiology at presentation			
NEWS (≤ / > 4)	75/96	12/39	0.009
Delirium (y/n)	14/158	12/36	0.001
Laboratory results at presentation			
CRP (< / ≥ 150 mg/L)	134/37	34/18	0.058
Albumin (≥ / < 35 g/L)	82/87	15/35	0.021
poGPS (0/1/2)	130/35/3	32/15/3	0.032
WCC (< 4.5 / ≥ 4.5—≤ 11.0/ > 11.0 × 10^9^/L)	23/118/30	7/34/11	0.750
Neutrophils (< / ≥ 7.5 × 10^9^/L)	132/39	34/18	0.088
Lymphocytes (≥ / < 1.5 × 10^9^/L)	52/118	7/45	0.015
NLR (< 3/ 3–5 / ≥ 5)	39/46/85	5/9/38	0.004
Hb (≥ / < 12.0 g/dL)	143/28	38/13	0.142
MCV (> / ≤ 99 fl)	149/21	40/11	0.102
Hct (male ≥ / < 0.40) (female ≥ / < 0.37) L/L	126/45	29/23	0.014
Platelets (≥ / < 150 × 10^9^)	140/30	40/12	0.383
Sodium (< 133/ ≥ 133– ≤ 146/ > 146 mmol/L)	17/153/2	9/40/3	0.013
Potassium (< 3.5/ ≥ 3.5– ≤ 5.5/ > 5.5 mmol/L)	13/142/2	5/36/0	0.822
Mg (≥ / < 0.75 mmol/L)	33/60	8/28	0.148
Urea (≤ / > 7.5 mmol/L)	125/47	24/28	< 0.001
Creatinine (≤ / > 130 umol/L)	159/13	43/9	0.039
AST (≤ / > 40 IU)	96/55	24/16	0.678
ALT (≤ / > 56 IU)	136/33	41/9	0.810
ALP (≤ / > 130 IU)	155/14	49/2	0.294
Bilirubin (≤ / > 17 mmol/L)	151/18	45/6	0.823
Glucose (≤ / > 7 mmol/L)	96/51	22/20	0.128
Lactate (< / ≥ 2 mmol/L)	43/21	17/12	0.426
HCO_3_ (≥ / < 22 mmol/L)	29/7	14/4	0.813
PT (≤ / > 13 s)	94/ 50	24/17	0.429
APPT (≤ / > 38 s)	133/8	36/3	0.642

Living circumstances: 0 = independent, 1 = sheltered accommodation, 2 = care home, 3 = nursing home

Ethnicity: White = 1, Mixed = 2, Asian = 3, Black = 4, Other ethnic groups or missing = 5

*poGPS* peri-operative Glasgow prognostic score, *NLR* neutrophil lymphocyte ratio

The median temperature of patients was 37.0 °C (IQR 36.3–38.0 °C). The majority of patients had a temperature < 37.5 °C (65%) and 14% of patients had a temperature < 36 °C. On admission the majority of patients had moderately severe or severe physiological derangement (NEWS score > 4) (60%) and had radiological changes characteristic of COVID-19 infection reported on chest X-ray (59%).

Of the laboratory analysis at presentation, the majority of patients had evidence of a systemic inflammatory response as evidenced by an elevated CRP > 80 mg/L (51%) and NLR (80%). The majority of patients had bilirubin (88%), alkaline phosphatase (91%), AST (54%), ALT (79%), glucose (53%), urea (67%), creatinine (90%), sodium (86%), potassium (80%), MCV (84%) and platelets (80%) within the laboratory reference range.

At 30-days following admission, 52 patients had died and the mortality rate was 23%. COVID-19 related respiratory failure (75%) and multiorgan failure (12%) were the commonest causes of death recorded. The relationship between 30-day mortality and clinicopathological characteristics are shown in Table 3. Death following admission for COVID-19 was associated with age ≥ 70 years (p < 0.001), past medical history of cognitive impairment (p ≤ 0.001), previous delirium (p < 0.001), clinical frailty score > 3 (p < 0.001), hypertension (p < 0.05), heart failure (p < 0.01), NEWS > 4 (p < 0.01), positive CXR (p < 0.01), and subsequent positive COVID-19 swab (p ≤ 0.001). Death was also associated with CRP > 80 mg/L (p < 0.05), albumin < 35 g/L (p < 0.05), poGPS (p < 0.05), lymphocytes < 1.5 10^9^/l (p < 0.05), neutrophil lymphocyte ratio (p ≤ 0.001), haematocrit (< 0.40 L/L (male)/ < 0.37 L/L (female)) (p ≤ 0.01), urea > 7.5 mmol/L (p < 0.001), creatinine > 130 mmol/L (p < 0.05) and elevated urea: albumin ratio (< 0.001).

To determine which admission parameters were independently associated with death within 30 days, binary logistic regression analysis was carried out (see Table 4). On analysis, age ≥ 70 years (O.R. 3.9, 95% C.I. 1.4–8.2, p < 0.001), past medical history of heart failure (O.R. 3.3, 95% C.I. 1.2–19.3, p < 0.05), NEWS > 4 at presentation (O.R. 2.4, 95% C.I. 1.1–4.4, p < 0.05), positive initial CXR (O.R. 0.4, 95% C.I. 0.2–0.9, p < 0.05) and poGPS (O.R. 2.3, 95% C.I. 1.1–4.4, p < 0.05) remained independently associated with death.

**Table 4:** Binary logistic regression analysis of clinicopathological characteristics of patients admitted with symptoms of COVID-19 (n = 224)

	Alive (n = 172)	Dead (n = 52)	p-value	O.R	95% CI	p-value
Age (< / ≥ 70 years)	124/48	19/33	< 0.001	3.9	1.4–8.2	< 0.001
Sex (male/female)	91/81	33/19	0.181			
BMI (< 20; ≥ 20–29; ≥ 30 kg/m^2^)	13/85/74	4/25/23	0.867			
SIMD (1 (most)–6 (least) deprived)	81/31/17/23/1/19	24/4/7/11/0/6	0.685			
Ethnicity (1–5)	159/0/7/2/3	50/0/0/0/2	0.774			
Living circumstances (0–3)	151/11/7/3	39/2/8/3	0.008			
Past Medical History						
Hypertension (y/n)	63/109	28/24	0.027	─	─	0.229
Heart failure (y/n)	12/160	11/41	0.003	3.3	1.2–19.3	0.028
T1DM (y/n)	2/170	0/52	0.436			
T2DM (y/n)	37/135	15/37	0.273			
Chronic renal failure (y/n)	18/154	10/42	0.095			
Cognitive impairment (y/n)	16/156	14/38	0.001			
Previous delirium (y/n)	6/166	10/40	< 0.001			
Frailty score (≤ / > 3)	107/65	14/38	< 0.001			
COPD (y/n)	29/143	12/40	0.311			
Smoker (never/ex/active)	82/70/20	24/27/1	0.428			
Alcohol excess (y/n)	22/150	9/43	0.410			
Liver disease (y/n)	15/157	4/48	0.816			
Hep C (never/previous/active)	167/2/2	51/1/0	0.650			
Active cancer (y/n)	6/166	3/49	0.464			
Diagnostic criteria						
PCR positive/Clinical Dx/Radiological Dx	74/7/91	37/1/14	0.001			
PCR negative/indeterminate/positive	43/42/83	4/9/39	0.001			
CXR negative/positive	63/107	30/21	0.006	0.40	0.4–0.9	0.020
Physiology at presentation						
NEWS (≤ / > 4)	75/96	12/39	0.009	2.4	1.1–4.4	0.024
Delirium (y/n)	14/158	12/36	0.001			
Laboratory results at presentation						
CRP (< / ≥ 150 mg/L)	134/37	34/18	0.058			
Albumin (≥ / < 35 g/L)	82/87	15/35	0.021			
poGPS (0/1/2)	130/35/3	32/15/3	0.032	2.2	1.1–4.4	0.024
WCC (< 4.5 / ≥ 4.5—≤ 11.0 / > 11.0 × 10^9^/L)	23/118/30	7/34/11	0.750			
Neutrophils (< / ≥ 7.5 × 10^9^/L)	132/39	34/18	0.088			
Lymphocytes (≥ / < 1.5 × 10^9^/L)	52/118	7/45	0.015			
NLR (< 3/ 3–5 / ≥ 5)	39/46/85	5/9/38	0.004	–	–	0.126
Hb (≥ / < 12.0 g/dL)	143/28	38/13	0.142			
MCV (> / ≤ 99 fl)	149/21	40/11	0.102			
Hct (male ≥ / < 0.40) (female ≥ / < 0.37) L/L	126/45	29/23	0.014	─	─	0.125
Platelets (≥ / < 150 × 10^9^)	140/30	40/12	0.383			
Sodium (< 133/ ≥ 133- ≤ 146/ > 146 mmol/L)	17/153/2	9/40/3	0.013			
Potassium (< 3.5/ ≥ 3.5- ≤ 5.5/ > 5.5 mmol/L)	13/142/2	5/36/0	0.822			
Mg (≥ / < 0.75 mmol/L)	33/60	8/28	0.148			
Urea (≤ / > 7.5 mmol/L)	125/47	24/28	< 0.001			
Creatinine (≤ / > 130 umol/L)	159/13	43/9	0.039			
AST (≤ / > 40 IU)	96/55	24/16	0.678			
ALT (≤ / > 56 IU)	136/33	41/9	0.810			
ALP (≤ / > 130 IU)	155/14	49/2	0.294			
Bilirubin (≤ / > 17 mmol/L)	151/18	45/6	0.823			

Living circumstances: 0 = independent, 1 = sheltered accommodation, 2 = care home, 3 = nursing home

Ethnicity: White = 1, Mixed = 2, Asian = 3, Black = 4, Other ethnic groups = 5

*poGPS* peri-operative Glasgow prognostic score, *NLR* neutrophil lymphocyte ratio

Among those patients who tested PCR COVID-19 positive (n = 122), age ≥ 70 years (O.R. 4.7, 95% C.I. 2.0–11.3, p < 0.001), past medical history of heart failure (O.R. 4.4, 95% C.I. 1.2–20.5, p < 0.05) and poGPS (O.R. 2.4, 95% C.I. 1.1–5.1, p < 0.05) remained independently associated with 30-days mortality (see Table 5).

**Table 5:** Binary logistic regression analysis of clinicopathological characteristics of patients admitted who had COVID-19 PCR + (n = 122)

	Alive (n = 83)	Dead (n = 39)	p-value	O.R	95%CI	p-value
Age (< / ≥ 70 years)	53/30	12/27	< 0.001	4.7	2.0–11.3	0.001
Sex (male/female)	39/44	16/23	0.219			
BMI (< 20; ≥ 20–29; ≥ 30 kg/m^2^)	5/37/41	3/18/18	0.798			
SIMD (1 (least)–6 (most) deprived)	9/1/16/10/21/37	4/0/9/6/2/21	0.959			
Ethnicity (1–5)	79/0/3/1/0	37/0/0/0/2	0.441			
Living circumstances (0–3)	72/8/2/1	30/0/7/2	0.027			
Past Medical History						
Hypertension (y/n)	33/50	20/19	0.233	–	–	0.765
Heart failure (y/n)	4/79	7/32	0.019	4.4	1.1–18.6	0.042
T1DM (y/n)	1/82	0/39	0.493			
T2DM (y/n)	22/61	10/29	0.920			
Chronic renal failure (y/n)	9/74	8/31	0.152			
Cognitive impairment (y/n)	8/75	12/27	0.003			
Previous delirium (y/n)	3/80	10/28	< 0.001			
Frailty score (≤ / > 3)	53/30	8/31	< 0.001			
COPD (y/n)	11/72	11/28	0.046	–	–	0.279
Smoker	41/35/7	16/22/1	0.833			
Alcohol excess (y/n)	5/78	5/34	0.204			
Liver disease (y/n)	6/77	2/37	0.663			
Hep C (never/previous/active)	82/0/1	39/0/0	0.493			
Active cancer (y/n)	4/79	1/38	0.560			
Diagnostic criteria						
CXR negative/positive	38/45	22/16	0.218			
Physiology at presentation						
NEWS (≤ / > 4)	36/46	10/29	0.054	─	─	0.146
Delirium (y/n)	6/77	10/27	0.003			
Laboratory results at presentation						
poGPS (0/1/2)	63/16/2	24/11/3	0.063	2.4	1.1–5.1	0.027
NLR (< 3/ 3–5 / ≥ 5)	21/23/38	5/6/28	0.015	─	─	0.144
Hb (≥ / < 12.0 g/dL)	65/17	28/10	0.497			
MCV (≤ / > 99 fl)	75/7	30/9	0.028	─	─	0.235
Hct (male ≥ / < 0.40) (female ≥ / < 0.37) L/L	59/23	23/16	0.155			
Platelets (< 150/ ≥ 150- < 450/ ≥ 450 × 10^9^)	14/67/0	9/30/1	0.452			
Sodium (≤ 133 /134 -145/ ≥ 146 mmol/L)	10/71/2	5/31/3	0.240			
Potassium (< 3.5/ ≥ 3.5 – ≤ 5.3/ > 53 mmol/L)	5/73/0	3/27/0	0.525			
Mg (≥ / < 0.75 mmol/L)	12/3	4/2	0.527			
Urea (≤ / > 7.0 mmol/L)	57/26	17/22	0.008			
Creatinine (≤ / > 130 umol/L)	76/7	34/5	0.450			
AST (≤ / > 40 IU)	48/26	21/28	0.466			
ALT (≤ / > 56 IU)	69/12	33/5	0.810			
ALP (≤ / > 150 IU)	75/6	37/1	0.304			
Bilirubin (≤ / > 17 mmol/L)	78/3	35/3	0.332			
Glucose (≤ / > 7 mmol/L)	40/27	18/12	0.978			
Lactate (< / ≥ 2 mmol/L)	24/6	13/7	0.241			
HCO_3_ (≥ / < 22 mmol/L)	16/4	9/3	0.744			
PT (≤ / > 13 s)	53/16	19/11	0.169			
APPT (≤ / > 38 s)	67/3	3/26	0.253			

Living circumstances: 0 = independent, 1 = sheltered accommodation, 2 = care home, 3 = nursing home

Ethnicity: White = 1, Mixed = 2, Asian = 3, Black = 4, Other ethnic groups = 5

*poGPS* peri-operative Glasgow prognostic score, *NLR* neutrophil lymphocyte ratio

## Discussion

The results of the present study show that, on admission and using routine clinical measures, there was a wide-ranging perturbance of clinicopathological parameters in patients with COVID-19. In particular, heart failure and activation of the systemic inflammatory response were independently associated with death at 30 days. Therefore, it would appear that, in addition to the current anti-viral therapeutic targets, the host systemic inflammatory response may be a legitimate therapeutic target in patients presenting to Emergency Departments with COVID-19.

The results of the present study are consistent with a number of other small studies [17–19] from Asia that have reported the usefulness of markers of the systemic inflammatory response to risk stratify patients with COVID-19. In particular, the NLR has been previously reported to have prognostic value in these patients [14, 20, 21]. For example, Liu et al. reported in a prospective validation study that NLR was a predictor of critical illness in 61 patients with PCR confirmed COVID-19 infection [21]. Similarly, Yang et al. reported in a randomly selected cohort of 69 non-severe and 24 severe cases that NLR > 3.3 was independently associated with “more severe illness” (HR 2.46; 95% CI 1.98–4.57; p < 0.05) [14]. Lagunas-Rangel reported a meta-analysis of NLR and lymphocyte-CRP ratio that included 5 studies from mainland China, comprising 828 patients, which concluded that increased NLR and low lymphocyte-CRP ratios, reflecting an enhanced inflammatory process, may suggest a poor prognosis [22]. The present study, compared with these previous studies, has greater detail in the admission clinicopathological characteristics, thereby reducing the potential confounding of unmeasured factors. Moreover, it uses a standardised measure of the systemic inflammatory response (developed to assess the magnitude of the post-operative systemic inflammatory response)—the poGPS [6]. This study is also among the first to provide data from a U.K. population.

**Fig. 1:**
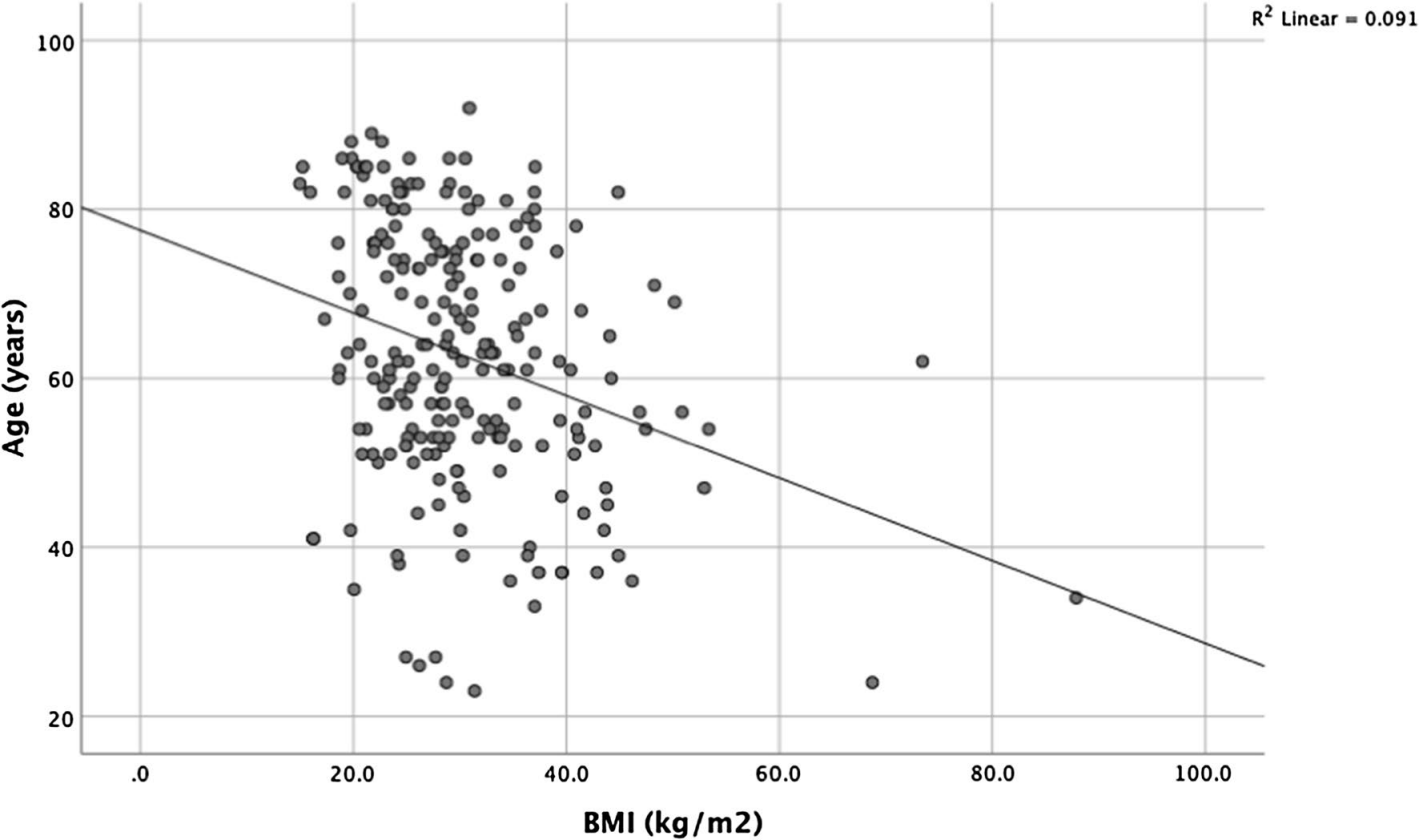
Relationship between BMI and age for patients admitted with COVID-19 (n = 244)

Data from the present study are also consistent with recent reports of an inverse relationship between age and BMI among patients with COVID-19 who were admitted to the intensive care setting (see Fig. 1) [23]. However, contrary to recent reports [24, 25], no significant association was found between BMI and mortality in the present study. Interestingly, a BMI of 40 kg/m^2^ or higher was identified by the Centre for Disease Control and Prevention in the USA as a factor that may increase a person's vulnerability to severe COVID-19 infection [26]. Nonetheless, despite the association between a BMI in the obese range and established risk factors for severe COVID-19 infection, such as type 2 diabetes, hypertension, and cardiovascular disease [25], data from the present study do not support an association with an increase in COVID-19 related mortality. The reasons for this are not clear. However, the present sample size may not have been large enough to detect the effect of obesity. Nevertheless, obesity compared with other physiological and inflammatory parameters is likely to have a smaller effect and may not provide a useful therapeutic target.

The post-operative systemic inflammatory response and the subsequent metabolic stress has been the subject of continuing interest over the last 40 years. In particular, there are well-developed therapeutic strategies to moderate the systemic inflammatory response. These include minimising surgical trauma and psychological distress; the use of anti-inflammatory agents (steroids) and antibiotics; fluid optimisation; optimal glucose control and nutritional support, to form an enhanced recovery strategy [7]. If the present results are confirmed, then it may be that using this knowledge to moderate the systemic inflammatory response associated with COVID-19 may reduce mortality. Indeed, the role of dexamethasone is endorsed by the recent positive report from the RECOVERY trial that showed a significant survival benefit at 28-days among patients who required either invasive mechanical ventilation or oxygen alone at randomization but not among those receiving no respiratory support [27]. Furthermore, there is emerging evidence of the importance of pro-inflammatory cytokines interleukin-6 (IL-6) and tumour necrosis factor (TNF-alpha) as predictors of mortality in patients with COVID-19 [28] and that IL-6 blockade appears to be beneficial [29]. Biran et al.have recently reported in a retrospective multi-centre observational study of 764 patients with severe SARS-CoV-2 infection requiring ICU support that treatment with a recombinant monoclonal antibody against the interleukin (IL)-6 receptor, Tocilizumab®, was associated with a 25% reduction in hospital-related mortality [29]. Randomised trials are required to confirm these results.

Despite the massive surge in COVID-19 related deaths, a relatively small proportion of the overall number infected have become unwell [1]. However, among those who do become unwell, clinical deterioration due to cytokine storm can occur with alarming rapidity, and mortality is high [18, 30]. The present results are consistent with these results and importantly offer a means of routine clinical assessment of an on-going systemic inflammatory response and its treatment since pro-inflammatory cytokine measurements are not routinely available from clinical laboratories.

Numerous randomised controlled trails of anti-viral agents are ongoing based on the premise that treating viral infection may benefit patients by reducing viral load and aiding recovery. To date, none of these studies have reported a significant mortality benefit. Therefore, in the absence of such direct intervention it may be important to minimise the systemic inflammatory response and support host metabolism in line with optimal peri-operative care. This strategy has the advantage of being part of routine clinical care and may complement more aggressive anti-viral strategies.

The present study has a number of limitations. There was a relatively small sample size and therefore subject to limitations such as sample bias. In addition, the ethnic background of the patients within this study was not as diverse as in other population centres globally. Furthermore, the level of deprivation was relatively high compared to other population centres in the UK. However, this study was based on routine clinical measurements and therefore readily subject to confirmation. Other less commonly utilised measurements, such as LDH and D-dimer have also been reported to have prognostic value in patients with COVID-19 infection [2]. However, these are recognised to be elevated as part of the systemic inflammatory response and may reflect the same process. Therefore, further work is required to rationalise the use of markers of the systemic inflammatory response in patients with COVID-19.

## Conclusion

Old age and severe systemic inflammation, as evidenced by the post-operative Glasgow Prognostic Score (poGPS), were independently associated with 30-day mortality in patients admitted to hospital with COVID-19 infection.

## Data Availability

Anonymized data will be made available on request to the corresponding author.

## Abbreviations

COVID-19: Novel corona virus-19
SIRS: Systemic inflammatory response
NEWS: National early warning score
PCR: Polymerase chain reaction
poGPS: Peri-operative Glasgow Prognostic Score
NLR: Neutrophil lymphocyte ratio
CRP: Serum C-reactive protein
GRI: Glasgow Royal Infirmary
ED: Emergency Department
AAU: Acute Assessment Unit
CXR: Chest X-ray
BMI: Body mass index
CFS: Clinical Frailty Scale
AVPU scale: Alert (A), Responding to verbal (V), painful (P) stimuli and unresponsive (U)
SIMD: Scottish Indices of Multiple Deprivation
χ^2^ test: Chi-squared test
